# Baricitinib as Salvage Therapy for Multidrug‐Resistant Chronic Graft‐Versus‐Host Disease

**DOI:** 10.1002/mco2.71094

**Published:** 2026-10-27

**Authors:** Zhuo‐Yu An, Chen Zhao, Yuanyuan Zhang, Xiaodong Mo, Leqing Cao, Daoxing Deng, Jun Kong, Fengrong Wang, Xiaojun Huang, Xiaohui Zhang

**Affiliations:** ^1^ Peking University People's Hospital Peking University Institute of Hematology National Clinical Research Center for Hematologic Disease Beijing Key Laboratory of Cell and Gene Therapy for Hematologic Malignancies Peking University Beijing China

1

Dear Editor:

Chronic graft‐versus‐host disease (cGVHD) remains the leading cause of late non‐relapse morbidity and mortality after allogeneic hematopoietic stem‐cell transplantation (allo‐HSCT). Ruxolitinib, a Janus kinase (JAK) 1/2 inhibitor, is a standard treatment for glucocorticoid‐refractory cGVHD, but grade ≥3 thrombocytopenia occurs in approximately 15% of treated patients and frequently forces dose reduction or discontinuation [1]. Patients who become ruxolitinib‐intolerant while their cGVHD remains active have few evidence‐based options and a poor prognosis, and salvage agents such as belumosudil are not universally available [2]. Such patients are typically heavily pre‐treated and at high risk, and options that control cGVHD without further aggravating cytopenias remain scarce. We therefore reviewed our single‐center experience with baricitinib in this difficult setting.

Baricitinib is an oral JAK1/JAK2 inhibitor. In preclinical models of GVHD, it both prevents and treats disease, increasing regulatory T cells through effects on JAK3 signaling and promoting intestinal epithelial repair via the epidermal growth factor receptor, which provides a mechanistic rationale for activity in cGVHD [3]. Because the principal clinical problem in ruxolitinib‐intolerant disease is myelosuppression, an agent with potentially less hematological toxicity is attractive; this rationale, however, has not been tested prospectively, and the present uncontrolled data cannot confirm it.

This report describes retrospective clinical observations made during routine care; it is not a prospective interventional study. We reviewed seven consecutive adults with cGVHD who were treated with baricitinib after allo‐HSCT at Peking University People's Hospital (Peking University Institute of Hematology) and who had discontinued ruxolitinib because of grade ≥3 thrombocytopenia (platelet count <50 × 10^9^/L). cGVHD was diagnosed and graded with the 2014 National Institutes of Health (NIH) Consensus Criteria [4], and treatment response was assessed with the 2014 NIH response criteria [5]. Full eligibility criteria, organ scoring, response definitions, concomitant immunosuppression, antiviral prophylaxis, statistical methods, and the ethics and consent statement are provided in the Supporting Information.

The cohort comprised six men and one woman (median age, 34 years; range, 30–53) who had undergone allo‐HSCT for hematologic malignancies (acute myeloid leukemia, acute lymphoblastic leukemia or lymphoma, myelodysplastic syndromes, and T‐ or NK/T‐cell lymphoma) from related donors (haploidentical, *n* = 4; matched or single‐allele‐mismatched sibling, *n* = 3). Patients had moderate (*n* = 1) or severe (*n* = 6) cGVHD involving a median of 3 organs (skin, *n* = 7; liver, *n* = 5; gastrointestinal tract, *n* = 2; eyes, *n* = 2; oral mucosa, *n* = 2; lung, *n* = 2; and joints/fascia, *n* = 1). Patients had received a median of 3 prior lines of systemic therapy (range, 2–4). All had been treated with ruxolitinib (median duration, 4 months) and developed grade ≥3 thrombocytopenia (median platelet nadir, 18 × 10^9^/L; range, 9–26), prompting discontinuation; the best response to ruxolitinib had been no response in five of seven patients and a partial response followed by loss of response in two of seven patients. Because platelet‐transfusion support and a variable washout interval preceded baricitinib, the platelet count at baricitinib initiation exceeded 50 × 10^9^/L in some patients. cGVHD severity at baricitinib initiation was equal to or greater than at ruxolitinib initiation in six of seven patients, indicating that the disease was genuinely ruxolitinib‐resistant rather than limited by toxicity alone.

The best overall response rate during follow‐up among patients receiving baricitinib (2–4 mg daily) was 86% (six of seven patients), comprising a complete response in four of seven (57%) and a partial response in two of seven (29%); the remaining patient had no documented response and ongoing active cGVHD. Platelet counts increased after baricitinib initiation in six of seven patients, with serial trajectories shown in Figure 1A. Responses spanned multiple organs—skin, liver, gastrointestinal tract, oral mucosa, and joints—with resolution of involved sites in the four patients achieving a complete response and partial improvement of skin, liver, and oral disease in the two with a partial response (Figure 1B); responses occurred early (documented time to response, 1–9 weeks). A corticosteroid‐sparing effect was also observed: five of seven patients (71%) discontinued corticosteroids, and the remaining two reduced their dose. Most responding patients remained on baricitinib with maintained responses at the most recent assessment. Because these are uncontrolled observations, they reflect associations and cannot establish a causal effect of baricitinib.

**FIGURE 1:**
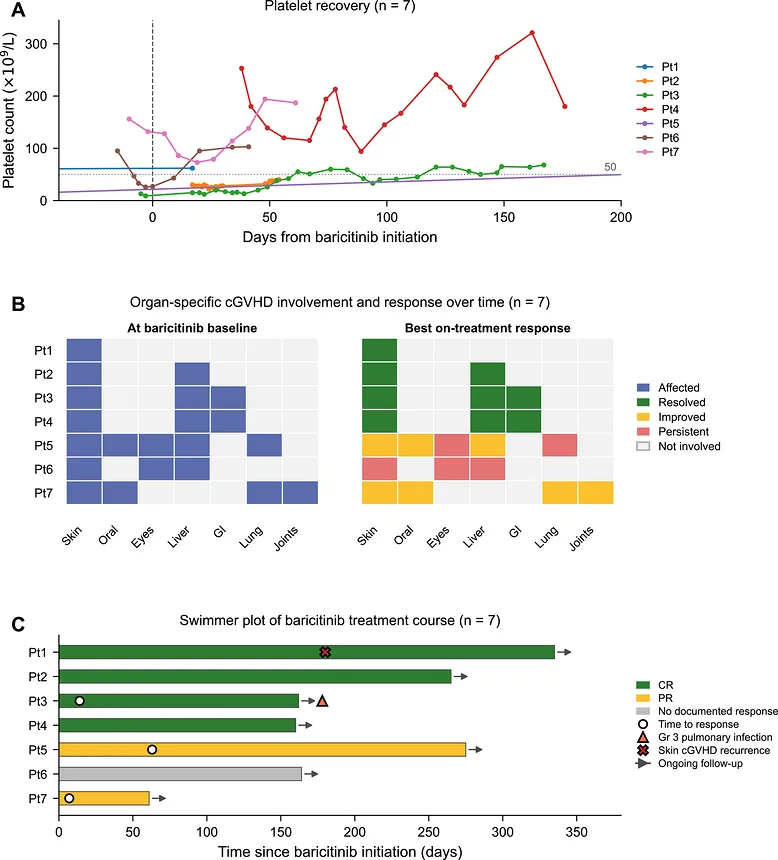
Hematological and clinical responses to baricitinib in seven patients with multidrug‐resistant chronic graft‐versus‐host disease (cGVHD). (A) Serial platelet counts for all seven patients plotted against time from baricitinib initiation (dashed vertical line); the dotted horizontal line marks 50 × 10^9^/L (*n* = 7). (B) Organ‐specific cGVHD involvement at baricitinib baseline (left) and best on‐treatment response (right) for each patient, by NIH organ site; best response is categorized as resolved, improved, or persistent (*n* = 7). (C) Swimmer plot of baricitinib treatment duration for all seven patients, colored by best overall response (complete response [CR], partial response [PR], or no documented response). Open circles denote the documented time to response, triangles denote grade 3 pulmonary infection, crosses denote skin cGVHD recurrence, and arrows indicate ongoing follow‐up (*n* = 7). Individual patient‐level values are shown in each panel. GI, gastrointestinal; NIH, National Institutes of Health; Pt, patient.

All seven patients received acyclovir prophylaxis according to institutional post‐transplant practice during baricitinib treatment. Baricitinib was generally well tolerated, and no patient discontinued treatment because of myelosuppression. One patient developed a grade 3 pulmonary infection that resolved with treatment. Cytomegalovirus or Epstein–Barr virus reactivation requiring pre‐emptive therapy occurred in three patients, in keeping with the expected post‐transplant course, and did not necessitate baricitinib discontinuation. No herpes‐zoster reactivation, thromboembolic events, major adverse cardiovascular events, or other opportunistic infections were observed. All seven patients were alive at last follow‐up (median, 5.4 months; range, 2.0–11.0), and the baricitinib treatment course for each patient is shown in Figure 1C.

In conclusion, in this small retrospective single‐center case series, baricitinib was associated with clinically meaningful organ responses, corticosteroid sparing, and platelet recovery in patients with severe, multidrug‐resistant, ruxolitinib‐intolerant cGVHD, and these observations suggest that baricitinib may be a feasible salvage option in this setting. The findings must be interpreted with caution because of the very small sample size, the absence of a control group, heterogeneous prior therapies and concomitant immunosuppression, the short and variable follow‐up, and the retrospective design, which together preclude attribution of the observed changes to baricitinib rather than to the natural disease course or co‐administered treatments. Prospective controlled studies are warranted to define the efficacy, optimal dosing, and long‐term safety of baricitinib after allo‐HSCT. A JAK inhibitor that preserves immunomodulatory activity while imposing less hematological toxicity would address a specific unmet need in ruxolitinib‐intolerant cGVHD, and this possibility merits prospective evaluation.

## Author Contributions

**Zhuo‐Yu An and Chen Zhao**: data curation; formal analysis; visualization; writing – original draft. **Yuanyuan Zhang, Xiaodong Mo, Leqing Cao, Daoxing Deng, Jun Kong, and Fengrong Wang**: investigation; writing – review and editing. **Xiaojun Huang and Xiaohui Zhang**: conceptualization; supervision; writing – review and editing. All authors have read and approved the final manuscript.

## Ethics Statement

This retrospective study was approved by the Ethics Committee of Peking University People's Hospital (approval No. 2025PHB197) and was conducted in accordance with the Declaration of Helsinki. Written informed consent for publication was obtained from all patients.

## Conflicts of Interest

The authors declare no conflicts of interest.

## Supporting information




**Supporting File**: mco271094‐sup‐0001‐SuppMat.docx

## Data Availability

The data that support the findings of this study are available from the corresponding author upon reasonable request.
