# Supplementary material for: Baricitinib as Salvage Therapy for Multidrug‐Resistant Chronic Graft‐Versus‐Host Disease
Source: MedComm (2020). 2026 Oct 27;7(11):e71094. doi: 10.1002/mco2.71094 (PMC13711017; doi:10.1002/mco2.71094)
Supplement: Supplementary file 1 [file MCO2-7-e71094-s001.docx]

**Baricitinib as Salvage Therapy for Multidrug-Resistant Chronic Graft-versus-Host Disease**

Zhuo-Yu An, M.D.#, Chen Zhao, M.D.#, Yuanyuan Zhang, M.D., Xiaodong Mo, M.D., Leqing Cao, M.D., Daoxing Deng, M.D., Jun Kong, M.D., Fengrong Wang, M.D., Xiaojun Huang, M.D., and Xiaohui Zhang, M.D.*

Peking University People’s Hospital, Peking University Institute of Hematology, National Clinical Research Center for Hematologic Disease, Beijing Key Laboratory of Cell and Gene Therapy for Hematologic Malignancies, Peking University, Beijing, China.

*Corresponding author: Xiaohui Zhang, M.D., zhangxh@bjmu.edu.cn, Peking University People’s Hospital, Peking University Institute of Hematology, Beijing, China. #These authors contributed equally.

**Supplementary Information**

**Supplementary Methods**

**Study design**

This study describes retrospective clinical observations made during routine care at a single centre; it was not a prospective interventional study.

**Case source**

All cases were identified at a single centre, Peking University People’s Hospital (Peking University Institute of Hematology), Beijing, China, a national referral centre for allogeneic haematopoietic stem-cell transplantation. No patients were recruited from community settings or other hospitals. Patients began baricitinib between August 2024 and July 2025.

**Inclusion criteria**

Adults were eligible if they had (i) chronic graft-versus-host disease (cGVHD) after allogeneic haematopoietic stem-cell transplantation; (ii) prior treatment with ruxolitinib; (iii) discontinuation of ruxolitinib because of grade ≥3 thrombocytopenia (platelet count <50 × 10⁹/L); (iv) subsequent treatment with baricitinib; and (v) available clinical follow-up. Seven consecutive patients met these criteria.

**Diagnosis and organ scoring**

cGVHD was diagnosed and graded according to the 2014 National Institutes of Health (NIH) Consensus Criteria, with organ-specific severity scored on the standard 0–3 scale and global severity classified as mild, moderate, or severe.

**Response assessment**

Treatment response was assessed using the 2014 NIH Response Criteria. Patients were evaluated at weeks 4, 8, and 12 after baricitinib initiation, where follow-up was available, and at the time of best response. Complete response (CR) denoted resolution of all reversible cGVHD manifestations; partial response (PR) denoted improvement in at least one organ without progression in others; the overall response rate combined CR and PR.

**Safety assessment**

Adverse events, infections (including viral reactivation), thromboembolic events, and cytopenias were recorded from the clinical record and graded according to the Common Terminology Criteria for Adverse Events (CTCAE), version 5.0.

**Concomitant immunosuppression and corticosteroid definition**

Concomitant immunosuppressive agents were continued or tapered at the discretion of the treating physician. Corticosteroid exposure is reported as the prednisone-equivalent dose in mg/kg/day; “pre-baricitinib” denotes the dose at baricitinib initiation and “last follow-up” denotes the dose at the most recent assessment or best response.

**Antiviral prophylaxis**

All seven patients received aciclovir prophylaxis according to institutional post-transplant practice during baricitinib treatment.

**Ethics and consent**

This study was approved by the Ethics Committee of Peking University People’s Hospital (approval No. 2025PHB197) and was conducted in accordance with the Declaration of Helsinki. Written informed consent for publication was obtained from all patients.

**Statistical analysis**

Analyses were descriptive. Continuous variables are summarised as the median and range and categorical variables as exact counts and proportions (numerator/denominator). No inferential statistical tests were performed, and no formal significance testing was undertaken given the small sample size; reported differences are descriptive only.
